# Oxidative Stress Alleviation by Sage Essential Oil in Co-amoxiclav induced Hepatotoxicity in Rats

**Published:** 2016-06

**Authors:** L. S. El-Hosseiny, N. N. Alqurashy, S. A. Sheweita

**Affiliations:** 1 Department of Environmental Studies, Institute of Graduate Studies and Research, Alexandria University, Egypt;; 2 Department of Biology, College of Science, Wasit University, Iraq;; 3 Department of Biotechnology, Institute of Graduate Studies and Research, Alexandria University, 163 El Horreya Avenue, P.O. box 832 El-Shatby, Alexandria 21526, Egypt

**Keywords:** Co-amoxiclav, hepatoprotection, oxidative stress, Sage essential oil

## Abstract

Clinical studies have shown that several classes of antibiotics are evidenced in drug induced liver injury. The combination of amoxicillin with clavulanic acid is commonly cited in such cases. Accordingly, the present study investigated the potential hepatoprotective and *in vivo* antioxidant efficacy of sage essential oil in Co-amoxiclav induced hepatotoxicity in rats. Sage essential oil was hydrodistilled from the aerial parts of *Salvia officinalis* L. and its compositional analysis was characterized by Gas chromatography-Mass spectroscopy. Rats were treated singly or concomitantly with Co-amoxiclav and sage essential oil for a period of seven days. The major components of sage oil as identified by GC-MS were 1,8-cineole, β-pinene, camphor, β-caryophyllene, α-pinene and α-caryophyllene comprising 26.3%, 14.4%, 10.9%, 7.8%, 6% and 2.5% respectively. The *in vivo* exposure of rats to Co-amoxiclav resulted in hepatotoxicity biochemically evidenced by the significant elevation of serum AST, ALT, ALP, γ-GT, total bilirubin and histologically conveyed by hydropic, inflammatory and cholestatic changes in rats’ liver. Oxidative stress mediated the hepatic injury as indicated by the significant escalation in lipid peroxidation, as well as, the significant depletion of both glutathione level and glutathione dependent enzymes’ activities. The concomitant administration of sage essential oil with Co-amoxiclav exerted a hepatoprotective effect via inducing an *in vivo* antioxidant defense response eventually regressing, to some extent, the hepatoarchitectural changes induced by Co-amoxiclav. Results suggest that sage essential oil is a potential candidate for counteracting hepatic injury associating Co-amoxiclav and this effect is in part related to the complexity of its chemical composition.

## INTRODUCTION

Medicinal plants have been priced for their medicinal, flavoring and aromatic qualities for centuries. As per the World Health Organization estimates, more than 60% of people in developing countries rely on plants for their primary health needs and a recent survey showed that more than 60% of patients use vitamins and phytomedicines in some stage of their therapy (1). Recently, it has been reported that nearly half of the agents used in liver therapy are either natural products or their derivatives (2). Sage plant is one of the widest break spread members of the Labiatae family and features prominently in the Pharmacopeias of many countries throughout the world. It has been used as a medication against perspiration and fever, as a carminative, spasmolytic, wound healing agent and in treating mental conditions (3). A range of the pharmacological properties of sage have been purported to its essential oil content (4).

Liver is an imperative organ that plays a crucial role in the metabolism of foreign substances rendering it not only the most important organ for detoxification but also a major target for their toxicity as well (5). Drug induced hepatotoxicity is one of the major concerns in medical practice and recent epidemiological data suggests that approximately 20 new cases of drug induced liver injury per 100000 persons occur each year. According to various registries and retrospective studies in European countries and the United States, antibiotics are the most common agents incriminated in the incidence of drug induced liver injury with Co-amoxiclav being cited as one of the leading causes of hospitalization for adverse hepatic events and accounting for 13-23% of drug induced hepatotoxicity cases (6-8).

Several lines of evidence suggest that oxidative stress plays a central role in the pathogenesis of drug induced liver injury and its induction significantly correlates with drug induced liver injury risk. In consensus with the resurgence of interest in natural products, screening of plants essential oils from chemical and pharmacological investigations to therapeutic aspects daunts researchers to visualize plants as potential sources of hepatoprotective agents. In view of the lacking evidence in relating the antioxidant competence of sage essential oil with hepatoprotective properties; the present study aimed at investigating the hepatoprotective and *in vivo* antioxidant potential of sage essential oil in Co-amoxiclav induced hepatotoxicity.

## MATERIALS AND METHODS

### Chemicals

Co-amoxiclav was obtained from GlaxoSmithKline®, Egypt. 5,5’-dithiobis nitro benzoic acid (DTNB), 1-chloro-2,4-dinitrobenzene (CDNB), oxidized glutathione (GSSG), NADPH, thiobarbituric acid (TBA) and cumene hydroperoxide were obtained from Sigma-Aldrich, St. Louis, MO, USA. All other reagents and solvents were of analytical grade.

### Plant material

*Salvia officinalis* L. (common sage) was obtained from the department of medicinal and aromatic plants, Horticulture Research Institute, Egypt.

### Extraction and characterization of sage essential oil

Dried aerial parts of sage were subjected to hydrodistillation for 4 hrs using Clevenger type apparatus (9). The obtained essential oil was collected, dried over anhydrous sodium sulphate and stored in sealed vials at 4°C. The chemical composition of the extracted oil was identified using thermo scientific GC-MS version 5 system. Five μL essential oil was diluted to 1mL with dichloromethane after which 2 μL was injected on splitless mode for 1min, followed by a split flow with a ratio of 1:10. The GC oven temperature was programmed from 60°C to 280°C at 3°C/min using helium as a carrier gas. Both the interface and injection temperatures were adjusted at 250°C. The ionization voltage was 70 eV with a mass range of 40-800 m/z. Identification of the oil constituents was done on the basis of their retention indices and by comparison of their mass spectral fragmentation patterns with those of MS library database (NIST and WILEY) (10). Quantitative analysis of each oil component, expressed in relative percentages of area, was carried out by peak area normalization measurements.

### Animals and treatment

Forty male albino rats weighing 150 ± 200 g obtained from the animal house of the Faculty of Medicine, Alexandria University were used for the current study. The local committee approved the experimental design and the protocol conforms to the guidelines of the National Institute of Health (NIH). The rats were housed in standard laboratory cages, under a 12-h light dark cycle at a constant ambient temperature (23°C) and humidity (30-60%) and were allowed free access to standard pellet diet and water *ad libitum.* After four weeks of acclimatization, the animals were assigned into four groups of ten rats each. The first group served as the control and received dimethyl sulphoxide as a vehicle, the second (Co-amoxiclav) group was given Co-amoxiclav orally at a dose of 30 mg/kg, the third (SEO) group was treated orally by sage essential oil dissolved in dimethyl sulphoxide at a dose of 0.052 ml/kg and the fourth group (SEO + Co-amoxiclav) received Co-amoxiclav and sage essential oil at the same dose and schedule as the second and third groups. After 7 consecutive days of treatment, rats were sacrificed and blood and liver specimens were collected. Blood samples were collected into plain vacutainer tubes and serum was obtained by centrifugation of blood at 3000 *xg* for 5 min. Liver tissues were washed with cold phosphate buffered saline and S9 fraction was prepared after homogenizing liver tissue in 0.01 M phosphate buffered solution (pH 7.4) and centrifuging at 10000*xg* for 20 min. Both serum and hepatic S9-fractions were stored at -80°C till subsequent analyses.

### Serum markers of hepatic toxicity

Liver function was analyzed by assessing serum aspartate aminotransferase (AST), alanine aminotransferase (ALT), alkaline phosphatase (ALP) and gamma-glutamyl transferase (γ-GT) activities, as well as, total bilirubin levels using Spectrum diagnostic kits (Spectrum®, Hannover, Germany). All analyses were done in duplicates based on the well-established spectrophotometric methods according to the kits' manuals supplied.

### Hepatic oxidative stress analysis

To examine the occurrence of oxidative damage in the liver, malondialdehyde (MDA) and reduced glutathione (GSH) levels, as well as, activities of the antioxidant enzymes; glutathione peroxidase (GPx), glutathione reductase (GR) and glutathione-S-transferase (GST) were assessed in hepatic S9 fraction of treated animals.

The extent of lipid peroxidation was estimated by determining the level of MDA using the method of Tappel and Zalkin (12). The content of GSH in liver was determined by the reaction with DTNB, according to the method of Mitchell *et al* (13). The activities of GPx, GR and GST were determined following the methods of Chiu *et al* (14), David and Richard (15) and Chi-Yu *et al* (16) respectively.

### Western blot analysis of hepatic GST and GPx

A total of 20 µg of protein per sample was separated on a 10% SDS-polyacrylamide gel and electroblotted on Hybond-C nitrocellulose membranes (Amersham, U.K.) Membranes were blocked with 5% BSA in Tris buffered saline and then probed with the primary antibodies for 3 h at room temperature. Antibodies used were specific monoclonal rabbit anti- GST π antibody or anti-GPx3 antibody (dilution 1:1000; Abcam UK). After incubation with primary antibody, the blot was washed three times in Tris buffered saline, followed by incubation for 30 min with anti-rabbit horseradish peroxidase IgG (dilution 1:10000; Bio Rad). The blot was then washed in Tris buffered saline, and antigen-antibody complexes were visualized after incubation for 1 min with enhanced luminescence reagent at room temperature, followed by exposure to Kodak XAR-5 film.

### Liver histological analysis

Slices of rats’ liver lobe were cleaned, dried and fixed in a solution of 10% buffered formalin. For histopathological examination, livers were processed by embedding in paraffin and sections were cut by rotatory microtome and mounted on glass slides. The sections were stained by conventional Hematoxylin and Eosin (H&E) stain and examined by light microscope (17).

### Statistical analysis

The data were expressed as mean ± standard error of the mean of n=10 rats per group. The statistical analysis was evaluated by one-way analysis of (ANOVA) using SPSS version 17 (SPSS, Inc, Chicago, IL) and the individual comparisons were done by Duncan’s multiple range test (DMRT). Values were considered statistically significant when *p*<0.05.

## RESULTS

**Figure 1:**
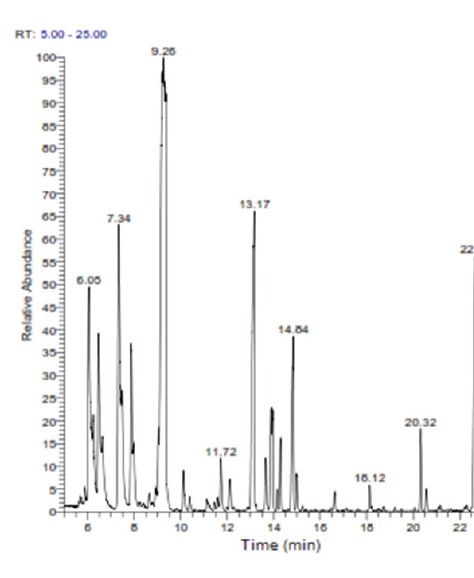
GC-MS chromatogram of the extracted sage essential oil.

The compositional analysis of the extracted sage oil by GC-MS revealed that 1,8-cineole, β-pinene and camphor were the major oil components constituting 26.28%, 14.41% and 10.85% of the total essential oil composition. Other chemical components constituting the hydrodistilled oil were the terpene hydrocarbons; α-pinene, α-caryophyllene and β- caryophyllene comprising 5.99%, 2.53% and 7.84% correspondingly. Moreover, the alcoholic terpene "borneol" and the ketonic terpene "thujone" constituted 3.57% and 1.76% of the extracted essential oil as shown in Figure 1 and Table 1.

**Table 1:** Chemical composition of the extracted sage essential oil as identified by GC-MS

Compound	Retention Time	Relative %

α-pinene	6.05	5.99
β-pinene	7.34	14.41
1,8-cineole	9.26	26.28
Thujone	11.72	1.76
Camphor	13.17	10.85
Borneol	14.84	3.57
Bornyl acetate	20.32	1.30
β-caryophyllene	22.67	7.84
α-caryophyllene	23.70	2.53

Treatment of rats with Co-amoxiclav for the designated period induced hepatic injury of mixed pattern, where both hepatocellular (AST and ALT) and hepatobiliary injury (ALP, γ-GT and total bilirubin) biomarkers significantly increased in Co-amoxiclav group. Meanwhile, hepatic injury was alleviated when sage essential oil was concomitantly administered with Co-amoxiclav as inferred by its restorative capacity on hepatic biomarkers to normal or near normal levels, although treatment of rats with sage essential oil alone insignificantly affected hepatic function biomarkers (Table 2).

**Table 2:** Changes in serum levels of hepatic biomarkers after treatment of rats with sage essential oil, Co-amoxiclav and their combination as a single daily dose for seven consecutive days

Parameter	Groups			
	Control	Co-amoxiclav	SEO	SEO + Co-amoxiclav

**AST (IU/L)**	99.4 ± 0.6^c^	106.1 ± 0.4^a^	101.8 ± 0.5^c^	103.1 ± 0.6^b^
**ALT (IU/L)**	80.9 ± 0.7^c^	106.6 ± 0.4^a^	81.9 ± 1.1^c^	99.1 ± 0.5^b^
**ALP (IU/L)**	152.5 ± 0.8^c^	185.7 ± 0.5^a^	159.3 ± 0.9^c^	163.4 ± 0.7^b^
**γ-GT (IU/L)**	2.28 ± 1.13^b^	3.5 5± 1.95^a^	2.14 ± 0.82^b^	2.06 ± 1.17^b^
**Total bilirubin (mg/dl)**	0.49 ± 0.06^b^	1.53 ± 0.07^a^	0.38 ± 0.12^b^	0.61 ± 0.11^b^

Values are expressed as mean ± SEM for 10 rats in each group.

a,b,c Values not sharing a common superscript letter in the same row differ significantly at *p*<0.05 (DMRT).

**Figure 2:**
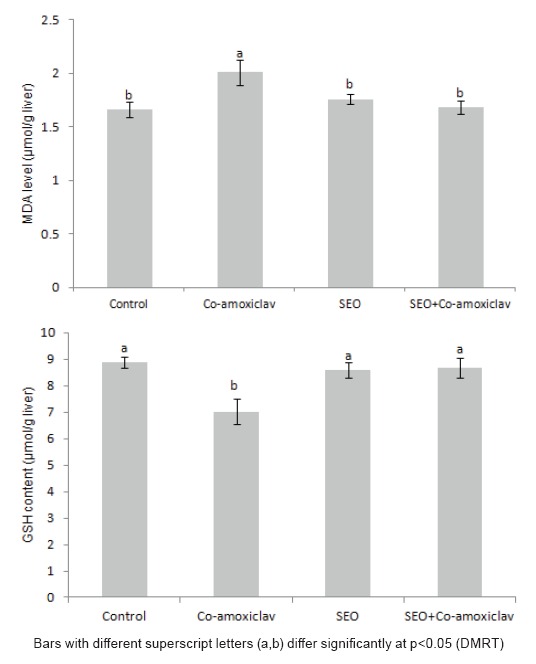
Changes in levels of malondialdehyde and reduced glutathione in livers of rats treated orally with Co-amoxiclav, sage essential oil and their combination.

**Figure 3:**
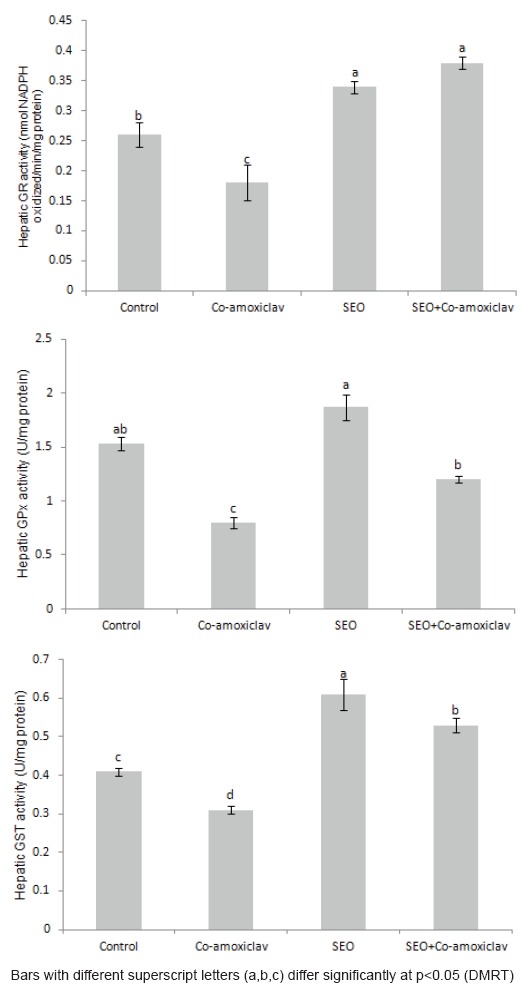
Changes in the activity of antioxidant enzymes in hepatic S9-fractions of rats treated orally with Co-amoxiclav, sage essential oil and their combination.

**Figure 4:**
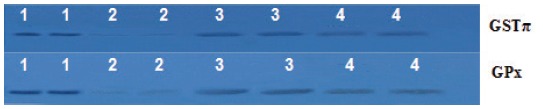
Western immunoblotting analysis showing protein expression of GST π and GPx3 isozymes. Lanes 1, 2, 3 and 4 represent the pooled protein of matched control, Co-amoxiclav, SEO and Co-amoxiclav+SEO treated rats.

The effect of sage essential oil and/or Co-amoxiclav on oxidative status of the liver varied. Exposure of rats to Co-amoxiclav significantly increased MDA level, a secondary product of lipid peroxidation, and significantly depleted GSH level, as well as, glutathione dependant enzymes activities (GR, GPx and GST) (Figures 2 and 3). Supporting the present perturbations in antioxidant defense parameters, protein expression of both GST and GPx was inhibited after treatment of rats with Co-amoxiclav (Figure 4). Sole treatment of rats with sage essential oil did not provoke any oxidative changes in the liver, where MDA level was insignificantly different from the control, however, the activities of glutathione dependant enzymes (GR, GPx and GST) increased significantly in sage essential oil group. Conversely, Co-administration of sage essential oil with Co-amoxiclav alleviated the oxidative damage induced by the antibiotic as conveyed by the decrease in escalated lipid peroxidation and compensation for the antioxidant deficits inflicted by Co-amoxiclav (Figures 2 and 3).

**Figure 5:**
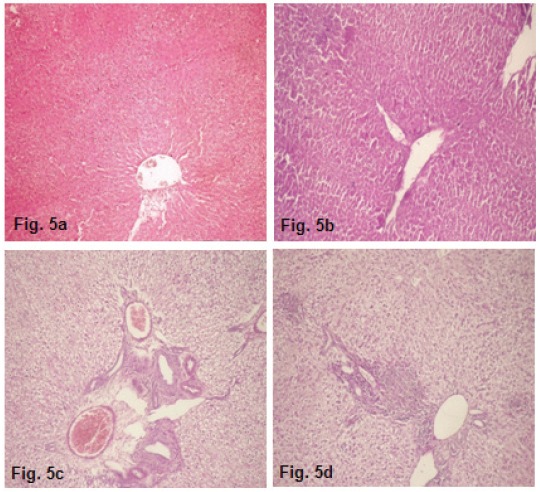
Photomicrograph of liver sections of rats from different treatment groups. (a) control (b) SEO (0.052 ml/kg BW) (c) Co-amoxiclav (30 mg/kg BW) (d) Co-amoxiclav+SEO (H&E stain ×200).

Histological examination of liver section of different treatment groups demonstrated that control rats exhibited normal hepatic architecture with no signs of inflammation, necrosis, fibrosis or cholestasis (Figure 5a). Likewise, liver of rats treated solely with sage essential oil did not exhibit any significant histological changes (Figure 5b). Rats' exposure to Co-amoxiclav provoked histoarchitectural changes manifested by hydropic changes, central vein necrosis and congestion, as well as, mild inflammatory and cholestatic changes (Figure 5c), whereas treatment of rats with sage essential oil in conjunction with Co-amoxiclav reversed, to some extent, the histopathological changes induced by Co-amoxiclav as is conveyed from the decrease in cholestatic changes and central vein congestion in liver of rats treated concomitantly with sage essential oil and Co-amoxiclav (Figure 5d).

## DISCUSSION

Essential oils are natural, complex multicomponent systems constituted by terpenes and their oxygenated derivatives. Many official monographs limit the content of essential oil, as well as, the specific limits of their major components as one of the characteristic specifications of these phytodrugs (18). In literature, the chemical composition of sage essential oils varies widely. The dominant constituents in many sage essential oils are *cis*-thujone (≤65.5%), 1,8-cineole (≤59%), camphor (≤45.7%), *trans*-thujone (≤40.1%), *α*-caryophyllene (33.7%) and linalool (≤35%). Furthermore, the German Drug Codex regulates the amounts of several constituents in sage essential oils as ≤20% for thujone, 14-37% for camphor, 6-16% for 1,8-cineole, ≤5% for borneol and ≤5% for bornyl acetate (19). In the present study, 1,8-cineole, camphor, thujone, α-pinene, β-pinene, α-caryophyllene, β-caryophyllene, borneol and bornyl acetate accounted for nearly 75% of the total sage essential oil composition. Qualitative rather than quantitative composition of the presently hydrodistilled sage oil is consistent with those described in other studies and entailed in some Pharmacopeias (18-20). The variability in chemical composition of essential oils amongst different studies is ascribed to several factors comprising genetic and environmental factors, climatic conditions in addition to extraction techniques (21).

Hepatotoxic compounds produce a wide variety of biochemical and histopathological outcomes and antibiotics in particular are associated with hepatic injury of different presentations and patterns (22). In the current investigation, the significant increments in the activities of serum AST and ALT, as well as, the concurrent increase in hepatobiliary injury biomarkers (ALP, γ-GT and total bilirubin) signify that Co-amoxiclav is a hepatotoxin, producing damage of mixed pattern (Table 2). Moreover, Co-amoxiclav exposure not only evoked hepatic injury but also induced oxidative stress in liver tissue, which was manifested by the significant elevation of MDA, as well as, the significant depletion in both non-enzymic (GSH) and enzymic antioxidants (GR, GPx and GST).

Changes in the activities of antioxidant enzymes can be considered as biomarkers of the antioxidant response (24). A common feature of most of the implicated enzymes is their function sequestering reactive oxygen species and/or maintaining the cell and cellular components in their appropriate redox state (24). Glutathione peroxidase catalyzes GSH oxidation to GSSG at the expense of H_2_O_2_ or other peroxides, and GR recycles oxidized glutathione back to reduced glutathione; therefore, their activities are essential for the intracellular quenching of cell damaging peroxide species and the effective recovery of the steady-state concentration of reduced glutathione (25). It has been reported that reactive oxygen species generation along with mitochondrial damage and intracellular glutathione depletion are the most important indicators of hepatotoxicity (26). Accordingly, in the current study, the decrease in the activity of antioxidant enzymes observed in Co-amoxiclav treated rats clearly indicates a negative response of the cell defense system to face oxidative insult induced and disruption of the antioxidant defense may account for the pathological process of exposure. Furthermore, the histopathological changes accrued in Co-amoxiclav treated group (Figure 5) may be related to the influence of oxidative stress and lipid peroxidation induction which disrupts membrane lipids inducing hydropic changes and eventually leads to leakage of liver enzymes into the blood stream. The presently evidenced biochemical and histological changes are in accordance with clinical studies in the context of hepatic damage pattern (27, 28) and are in line with pharmacovigilance studies reporting that hepatotoxicity prevalence is higher for Co-amoxiclav rather than amoxicillin alone (29, 30). Moreover, occasioning of oxidative stress in Co-amoxiclav treated rats is consistent with previous research reports contesting the main role of oxidative stress in cytological injury induced by Co-amoxiclav (31-33).

Natural antioxidants specially phytochemicals have gained popularity worldwide and have been proposed as agents to counteract liver damage (34). In the current study, sage essential oil exhibited an *in vivo* antioxidant potential against Co-amoxiclav induced oxidative stress implicated in hepatic injury induced by the antibiotic. As depicted in Figure 2, the escalated lipid peroxidation was restored to normal levels and the depleted non-enzymic (GSH) and enzymic (GST, GPx and GR) antioxidants were significantly increased in rats treated concurrently with sage essential oil and Co-amoxiclav. The *ex vivo* antioxidant potential of sage has been documented in literature (3), however few *in vivo* studies addressed the antioxidant potential of sage essential oil or sage extracts. The cellular antioxidant activity of *Salvia officinalis* has been demonstrated on freshly isolated rat hepatocytes, where sage essential oil showed a protective effect against the oxidative compound *tert-*butyl hydroperoxide (35). Another study revealed an antioxidant capacity for sage tea on rat hepatocytes in primary culture and for sage methanolic extract against oxidative damage induced by *tert-*butyl hydroperoxide on HepG2 cells (35, 36). Taking into account the present results, it can be conceived that sage essential oil evoked an antioxidant effect against Co-amoxiclav induced oxidative stress as evidenced by the significant decrease in escalated lipid peroxidation and compensation for endogenous antioxidant deficits. Intriguingly, when sage essential oil was administered to rats alone, it resulted in a significant increase in the activities of antioxidant enzymes without inducing lipid peroxidation. A noteworthy feature of the biological system is its heterogeneity, where the antioxidants are localized in the aqueous and lipid phase of the cell. The lipophilic antioxidants are distributed in the lipophilic compartments, whereas the hydrophilic antioxidants reside and scavenge radicals in the aqueous phase of the cell (37). In this context, sage essential oil is privileged with the lipophilic characteristic that gives it advantage over aqueous or alcoholic sage extracts. The endowed lipophilicity and antioxidant capacity of essential oils probably enhances their distribution in lipophilic compartments of the cells retarding peroxidative reactions and augmenting the role of endogenous antioxidant defense system.

The chemical composition of essential oils and the variety of chemical structures of their constituents are responsible for a wide range of biological activities many of which are of significance in different contexts of human health (38). Given the complexity of their chemical composition, it is suggested that their mode of action is complex, and it is difficult to rule out the chemical constituent that contributes to their biological activity or to identify the molecular pathway of their action. The hepatoprotective activity exerted by the presently investigated essential oil may be attributed to 1,8-cineole, as the major component of sage essential oil. This monoterpene was reported to have various pharmacological effects including anti-inflammatory, antioxidant and antinociceptive activities (39). In an *in vivo* murine model of septic shock that is characterized by lysis of hepatocytes, 1,8-cineole suppressed the elevation in serum transaminase activity and prevented the necrosis and haemorrhage associating the septic shock to an extent greater than dexamethasone (40). The mono terpenoid, 1,8-cineole was also reported to exert a hepatoprotective effect via activating the antioxidant defense system against the oxidative damage induced by the environmental contaminant 2,3,7,8-tetrachlorodibenzo-*p*-dioxin (41). Besides 1,8-cineole, an *in vivo* antioxidant capacity was also reported for β-pinene (42), a monoterpene that is present in the currently investigated sage oil in a relatively high amount (14.4%) implying that the major components constituting essential oils may contribute to their exhibited biological activities.

## CONCLUSION

Conclusively, the present results demonstrated that administration of sage essential oil exerted beneficial effects in alleviating Co-amoxiclav induced hepatotoxicity in rats by limiting the extent of lipid peroxidation and hence cell membrane injuries. Considering the significant impact of sage on the examined antioxidant enzymes, it can be proposed that sage essential oil mediates its hepatoprotective effect through activation of antioxidant defense mechanisms. Meanwhile, the contribution of various essential oil components to the effect exerted by sage essential oil and their specific mechanism of action are still to be elucidated.

## References

[1] Govind P (2011). Medicinal plants against liver diseases. IRJP.

[2] Zhang A, Sun H, Wang X (2013). Recent advances in natural products from plants for treatment of liver diseases. Eur. J. Med. Chem.

[3] Baricevic D, Bartol T, Kintzios SE (2000). The biological/pharmacological activity of Salvia genus. “Sage-The genus Salvia”, Amesterdam, Hardwood Academic Publishers.

[4] Miguel MG (2010). Antioxidant and anti-inflammatory activities of essential oils: a short review. Molecules.

[5] Ghabril M, Chalasani N, Bjornsson E (2010). Drug-induced liver injury: a clinical update. Curr. Opin. Gastroenterol.

[6] Leise MD, Poterucha JJ, Talwalkar JA (2014). Drug-induced liver injury. Mayo clinic proceedings.

[7] Andrade RJ, Tulkens PM (2011). Hepatic safety of antibiotics used in primary care. J. Antimicrob. Chemother.

[8] Salvo F, Polimeni G, Moretti U, Conforti A, Leone R, Leoni O (2007). Adverse drug reactions related to amoxicillin alone and in association with clavulanic acid: data from spontaneous reporting in Italy. J. Antimicrob. Chemother.

[9] European Pharmacopoeia A common European initiative by the Council of Europe. Chimia-Zurich.

[10] Adams RP (2007). Identification of essential oil components by gas chromatography/mass spectrometry.

[11] Opdyke DL (1974). Monographs on fragrance raw materials. Food Cosmet. Toxicol.

[12] Tappel AL, Zalkin H (1959). Inhibition of lipid peroxidation in mitochondria by vitamin E. Arch. Biochem. Biophys.

[13] Mitchell JR, Jollow DJ, Potter WZ, Davis DC, Gillette JR, Brodie BB (1973). Acetaminophen-induced hepatic necrosis. I. Role of drug metabolism. J. Pharmacol. Exp. Ther.

[14] Chiu DT, Stults FH, Tappel AL (1976). Purification and properties of rat lung soluble glutathione peroxidase. Biochim. Biophys. Acta.

[15] David M, Richard JS, Bergmeyer J, Marianna GB (1983). Methods of enzymatic analysis.

[16] Chi-Yu L, Lynt J, Richard HC, James DM (1981). Mouse liver glutathione-S-transferase. J. Biol. Chem.

[17] Drury AR, Wallington EA (1980). Carleton’s histological techniques.

[18] Aromadee C, Salih B, Celikbicak O (2012). The qualitative and quantitative determinations of volatile constituents in some herbal medicines by gas chromatography. Gas chromatography in plant science, wine technology, toxicology and some specific applications.

[19] Abu-Darwish MS, Cruz MT, Cabral C, Al-Bdour TH (2013). Essential oil of common sage (*Salvia officinalis* L.) from Jordan: assessment of safety in mammalian cells and its antifungal and anti-inflammatory potential. Biomed. Res. Int.

[20] Arraiza MP, Arrabal C, Lopez JF (2012). Seasonal variation of essential oil yield and composition of sage (*Salvia officinalis* L.) grown in Castilla - La Mancha (Central Spain). Not. Bot. Horti Agrobot.

[21] Ben-Farhat MB, Jordan MJ, Chaouech-Hamada R, Landoulsi A (2009). Variations in essential oil, phenolic compounds, and antioxidant activity of tunisian cultivated *Salvia officinalis* L. J. Agric. Food Chem.

[22] Leitner MJ, Graninger W, Thalhammer F (2004). Hepatotoxicity of antibacterials: Pathomechanisms and clinical data. Infection.

[23] Alia M, Ramos S, Mateos R, Bravo L (2006). Quercetin protects human hepatoma cell line (HepG2) against oxidative stress induced by *tert*-butyl hydroperoxide. Toxicol. Appl. Pharmacol.

[24] Valko M, Leibfritz L, Moncol J, Cronin MT (2007). Free radicals and antioxidants in normal physiological functions and human disease. Int. J. Biochem. Cell Biol.

[25] Townsend DM, Manevich Y, He L, Hutchens S (2009). Novel role for glutathione- S-transferase π. J. Biol. Chem.

[26] Singh A, Bhat T, Sharma OP (2011). Clinical biochemistry of hepatotoxicity. J. Clin. Toxicol.

[27] (2013). GlaxoSmithKline, Clavulin® product monograph, Antibiotic and β-lactamase inhibitor.. Submission Control No. 164138.

[28] Jordan T, Gonzalez M, Casado M, Suarez JF (2002). Amoxicillin clavulanic acid induced hepatotoxicity with progression to cirrhosis. J. Gastroenterol. Hepatol.

[29] Bush K, Finch RG, Greenwood D, Norrby SR, Whitley RJ (2003). β-lactam antibiotics: Penicillin and other β-lactam antibiotics. Antibiotic and chemotherapy: anti-infective agents and their use in therapy.

[30] GlaxoSmithKline, 2009, Amoxil® (Amoxycillin) product monograph North Carolina US. http://us.gsk.com/products/assets/us_amoxil.pdf.

[31] El-Sherbiny GA, Taye A, Abdel-Raheem IT (2009). Role of ursodeoxycholic acid in the prevention of hepatotoxicity caused by amoxicillin-clavulanic acid in rats. Ann. Hepatol.

[32] Olayinka ET, Olukowade IL (2010). Effect of amoxycillin/clavulanic acid (Augmentin 625®) on antioxidant indices and markers of renal and hepatic damage in rats. Toxicol. Environ. Health Sci.

[33] Olayinka ET, Olukowade IL, Oyediran O (2012). Amoxycillin/clavulanic acid combinations (Augmentin® 375 and 625® tablets) induce - oxidative stress, and renal and hepatic damage in rats. AJPP.

[34] Vitaglione P, Morisco F, Caporaso N, Fogliano V (2004). Dietary antioxidant compounds and liver health. Crit. Rev. Food Sci. Nutr.

[35] Lima CF, Valentao PCR, Andrade RB, Seabra RM (2007). Water and methanolic extracts of *Salvia officinalis* protect HepG2 cells from *t*-BHP induced oxidative damage. Chem. Biol. Interact.

[36] Lima CF, Andrade RB, Seabra RM, Fernandes-Ferreira M (2005). The drinking of a Salvia officinalis infusion improves liver antioxidant status in mice and rats. J. Ethnopharmacol.

[37] Niki E, Yoshida Y, Saito Y, Noguchi N (2005). Lipid peroxidation: mechanisms, inhibition and biological effects. Biochem. Biophys. Res. Commun.

[38] Bakkali F, Averbeck S, Averbeck D, Idaomar M (2008). Biological effects of essential oils-A review. Food Chem. Toxicol.

[39] Koziol A, Stryjewska A, Librowski T, Salat K (2014). An overview of the pharmacological properties and potential applications of natural monoterpenes. Mini Rev. Med.Chem.

[40] Santos FA, Silva RM, Tomé AR, Rao VS (2001). 1,8-cineole protects against liver failure in an *in-vivo* murine model of endotoxemic shock. J. Pharm. Pharmacol.

[41] Ciftci O, Ozdemir I, Tanyildizi S, Yildiz S (2011). Antioxidative effects of curcumin, β-myrcene and 1,8-cineole against 2,3,7,8-tetrachlorodibenzop-dioxin-induced oxidative stress in rats liver. Toxicol. Ind. Health.

[42] Wang SY, Chen CT, Sciarappa W, Wang CY, Camp MJ (2008). Fruit quality, antioxidant capacity, and flavonoid content of organically and conventionally grown blueberries. J. Agric. Food Chem.

